# Low resolution thermal imaging dataset of sign language digits

**DOI:** 10.1016/j.dib.2022.107977

**Published:** 2022-02-23

**Authors:** Sreenivasa Reddy Yeduri, Daniel Skomedal Breland, Simen Birkeland Skriubakken, Om Jee Pandey, Linga Reddy Cenkeramaddi

**Affiliations:** a Department of Information and Communication Technology, University of Agder, Norway; b Department of Electronics Engineering, IIT BHU Varanasi, Varanasi 221005, India

**Keywords:** Thermal imaging, Sign language digits, Thermal camera, Machine learning models, Sensor, Temperature

## Abstract

The dataset contains low resolution thermal images corresponding to various sign language digits represented by hand and captured using the Omron D6T thermal camera. The resolution of the camera is 32×32 pixels. Because of the low resolution of the images captured by this camera, machine learning models for detecting and classifying sign language digits face additional challenges. Furthermore, the sensor’s position and quality have a significant impact on the quality of the captured images. In addition, it is affected by external factors such as the temperature of the surface in comparison to the temperature of the hand. The dataset consists of 3200 images corresponding to ten sign digits, 0–9. Thus, each sign language digit consists of 320 images collected from different persons. The hand is oriented in various ways to capture all of the variations in the dataset.

**Specifications Table**

This section list the details of the hardware, procedure for collecting the data, and the format of the data.

**Table:** 

Subject	Human-Computer Interaction, Biomedical, Electrical and Electronic Engineering
Specific subject area	Thermal images of different sign language digits represented using hand
Type of data	Image (.png)
How data were acquired	Thermal Camera (Omron D6T module)
	Camera Stand
	Raspberry Pi 3 Model B
Data format	Raw (from acquisition)
Parameters for data collection	Images are collected from 32 people with 32×32 pixel camera for different signs with different hand orientations
Description of data collection	It is hard to capture good images with an unstable low resolution camera. Thus, the camera is placed on a flexible stand to move and fix the stand based on the position of the hand. The software program was designed to save images based on the number that is being pressed as input in the range 0 to 9. For example, a number 2 is pressed on the keyboard to capture the thermal image corresponding to digit 2.
Application scenario	Human-computer interaction, industrial robotics, and automotive user interfaces
Data source location	ACPS group, Department of Information and Communication Technology, University of Agder, Grimstad, Norway
Data accessibility	Repository Name:
	thermal_image_dataset
	https://github.com/ysrysr117/Thermal-Image-Dataset
	https://doi.org/10.5281/zenodo.6053169
Related research article	D. S. Breland, S. B. Skriubakken, A. Dayal, A. Jha, P. K. Yalavarthy, L. R. Cenkeramaddi, Deep learning-basedsign language digits recognition from thermal images with edge computing system, IEEE Sensors Journal 21 (2021)10445-10453.

## Value of the Data

• The dataset is useful for developing novel machine learning algorithms for efficient sign language digit classification.
• The academic or research communities working on thermal imaging data with efficient machine learning algorithms for sign language digit classification.
• The data is useful for developing and testing novel algorithms to work on thermal imaging dataset.
• The data is collected without any constraints on the environment as well as the data has been captured with low resolution camera. This in turn, more useful for testing the algorithms with thermal imaging data.
• The data is collected with different hand orientations to incorporate all variations in the dataset. As most of the persons are right-handed, we created the dataset with right hand.

## 1 Data Description

The dataset contains the images captured from low resolution thermal camera. The images are captured from random people for different sign language digits ranging from 0 to 9. We also consider different hand orientations while capturing the images. We have divided the total dataset into three parts such as training, validation and testing. The 80% of the data for training, 10% of the data for validation and the remaining, 10% is used for testing.

### 1.1 Data file description

**Fig. 1:**
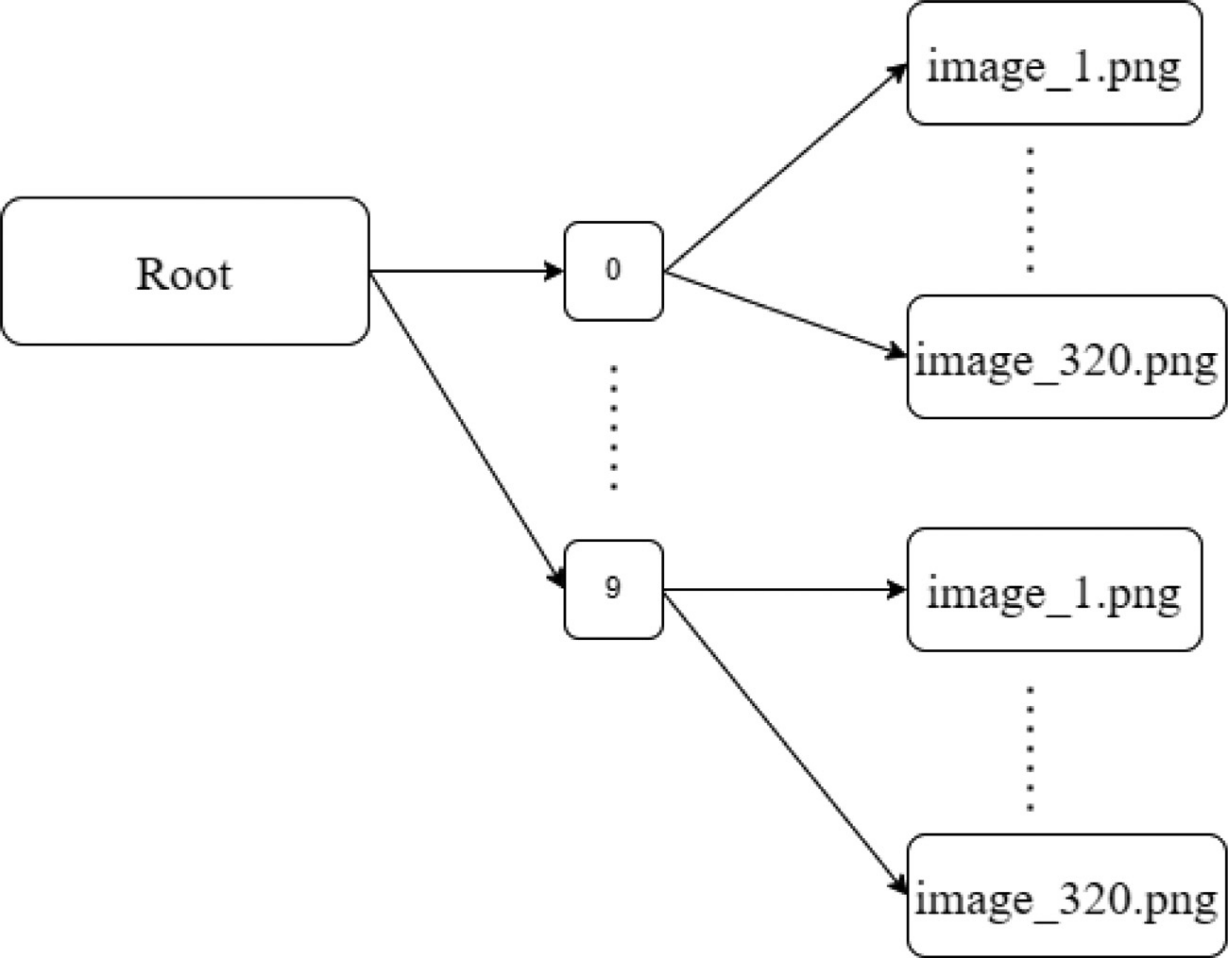
Data structure of the repository.

Fig. 1 shows the structure of the data repository. The root folder consists of ten folders namely 0 to 9. Each folder consists of 320 images in.png format. These images are captured from 32 people with different hand orientations. The total size of the dataset is 8.20 MB [1].

**Fig. 2:**
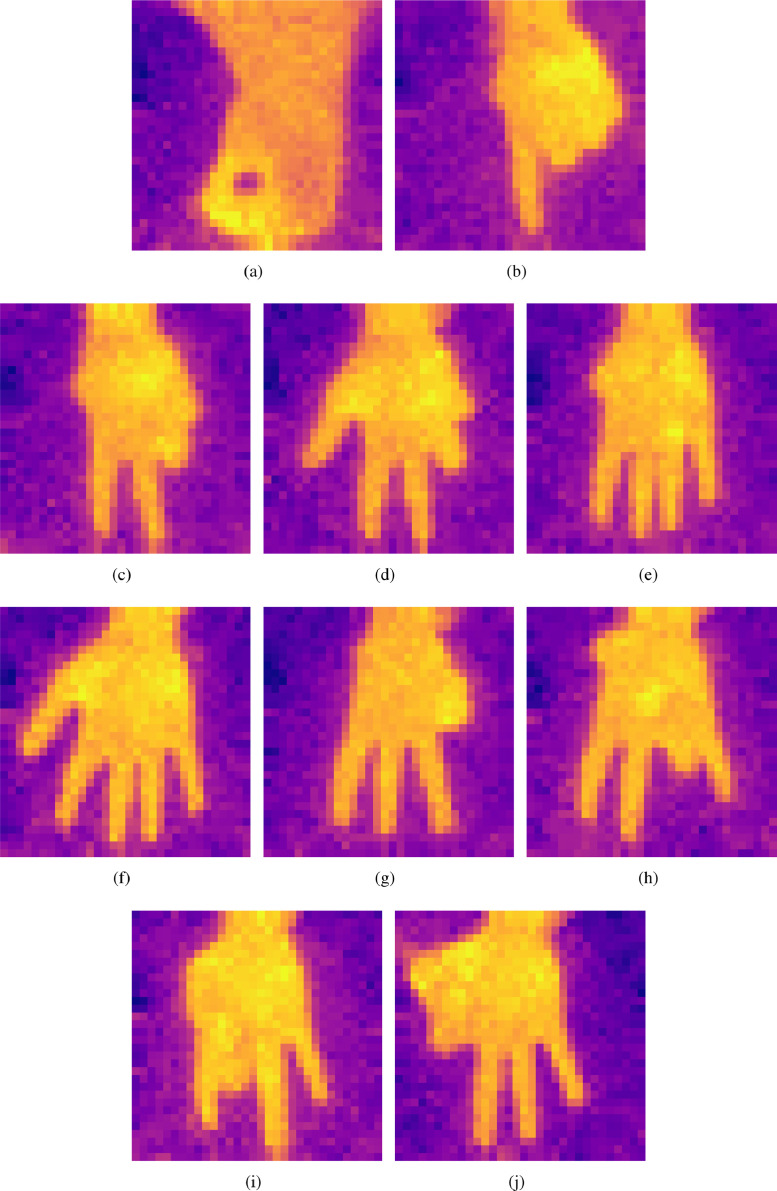
A thermal image corresponding to: (a) Digit 0; (b) Digit 1; (c) Digit 2; (d) Digit 3; (e) Digit 4; (f) Digit 5; (g) Digit 6; (h) Digit 7; (i) Digit 8; and, (j) Digit 9.

Fig. 2 shows the thermal images corresponding to digits 0 to 9. Fig. 2(a) is corresponding to digit 0 and Fig. 2(b) corresponds to digit 1. Figs. 2(c), 2(d), 2(e), 2(f), 2(g), 2(h), 2(i), and 2(j) are the thermal images corresponding to digits 2, 3, 4, 5, 6, 7, 8, and 9, respectively.

**Fig. 3:**
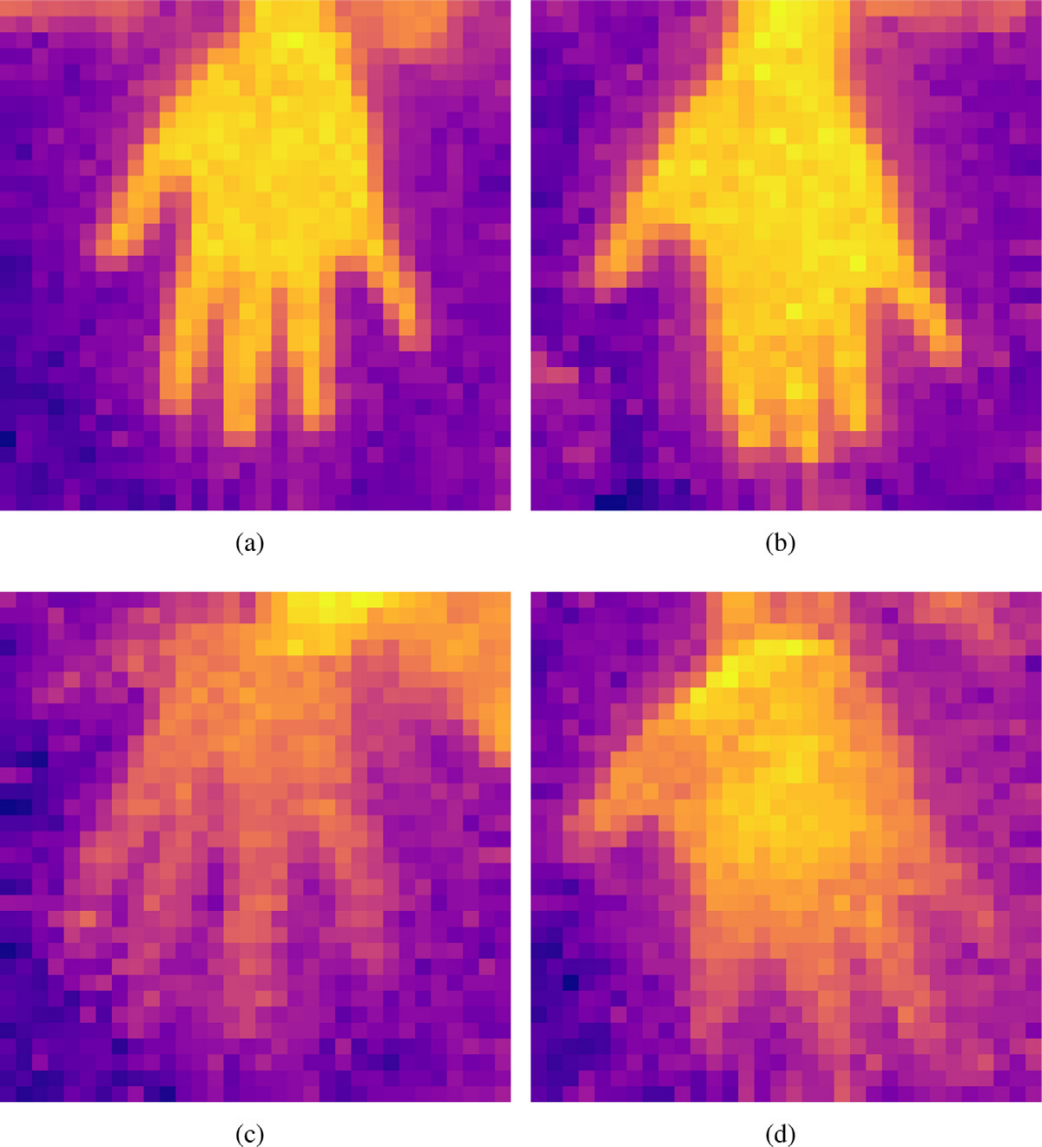
A thermal image with (a) Good quality and proper hand orientation; (b) Medium quality and improper orientation; (c) Poor quality and good orientation; and, (d) Varying quality from hand-palm to fingers.

Fig. 3 shows the example images in the data repository corresponding to digit 5 with different qualities. Fig. 3(a) shows a thermal image with good due to the proper positioning of the hand. Fig. 3(b) shows the thermal image with medium quality due to the different orientation.

Fig. 3 (c) shows the poor quality image with good positioning and the image with poor quality and improper orientation is shown in Fig. 3(d). Thus, the dataset contains the images from low quality to high quality which gives more challenge to the algorithms working on thermal imaging data.

## 2 Experimental Design, Materials and Methods

**Fig. 4:**
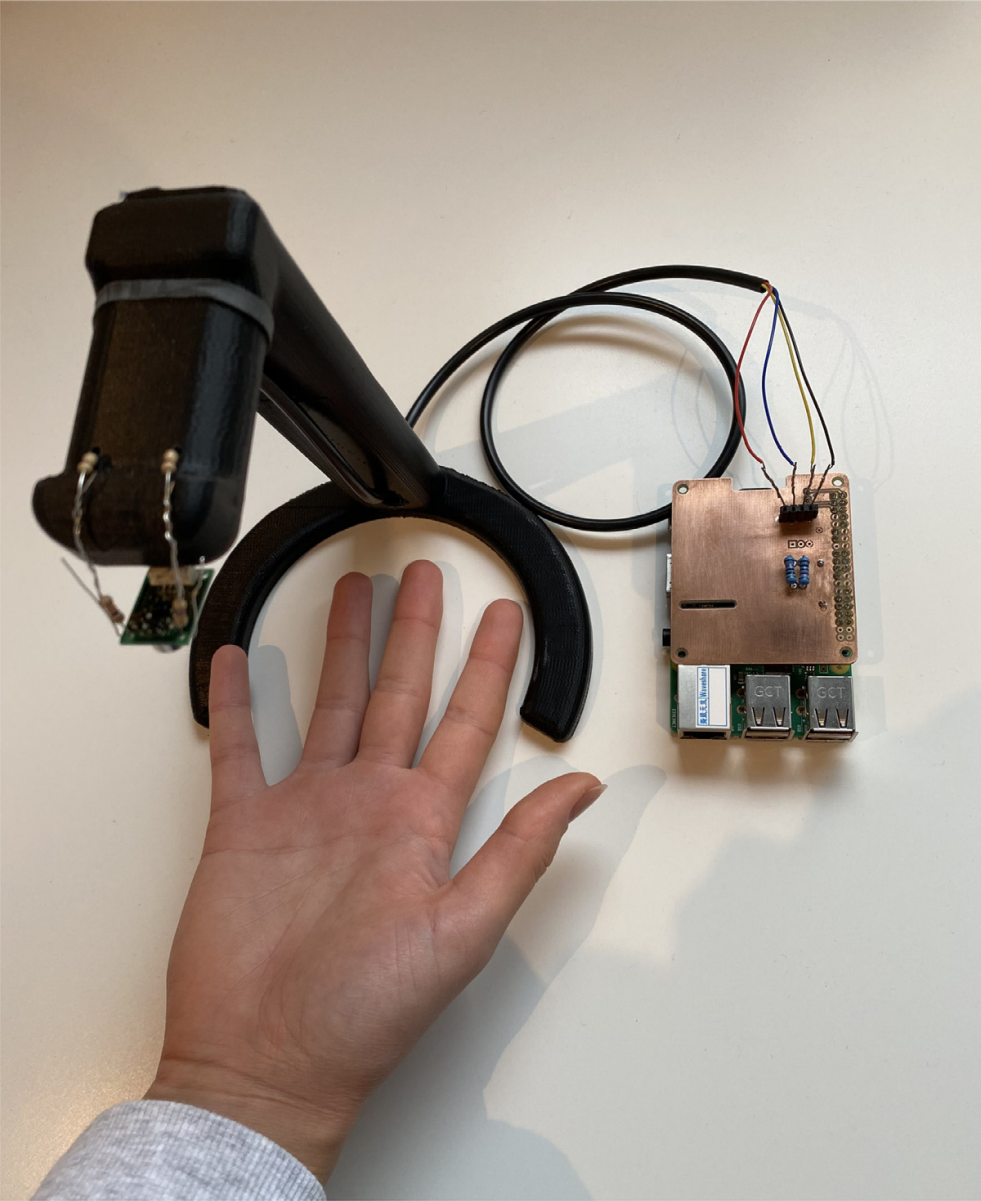
Thermal camera setup for the collection of sign language digits from right hand.

Fig. 4 shows the experimental setup of the thermal camera considered for the data collection. We consider thermal camera Omron D6T module, a camera module designed to save space to be fitted in embedded systems [2]. As the calculation is done within the camera module, it reduces the overall computational complexity. As the product sheet describes, it uses a micro electromechanical system (MEMS) thermal sensor which is low cost with high accuracy. The thermal image from this module is 32×32 pixels which is common in most of the D6T family. The exact name of this module is D6T-32L-01A, with a square image and Field of View (FOV) of $90^{\circ }$. For example, when this thermal camera situated at one meter distance, it can capture up to two meters in both x and y direction. It has a temperature detection range of $0^{\circ }$C to $200^{\circ }$C of objects and ambient temperature detection range of $0^{\circ }$C to $80^{\circ }$C. The D6T module is attached to a mounted stand for stabilization [3]. With the low resolution, it would be very hard to produce good images if the camera were not stabilized. The stand was made to take images down towards a surface, where people would place their hands.

**Fig. 5:**
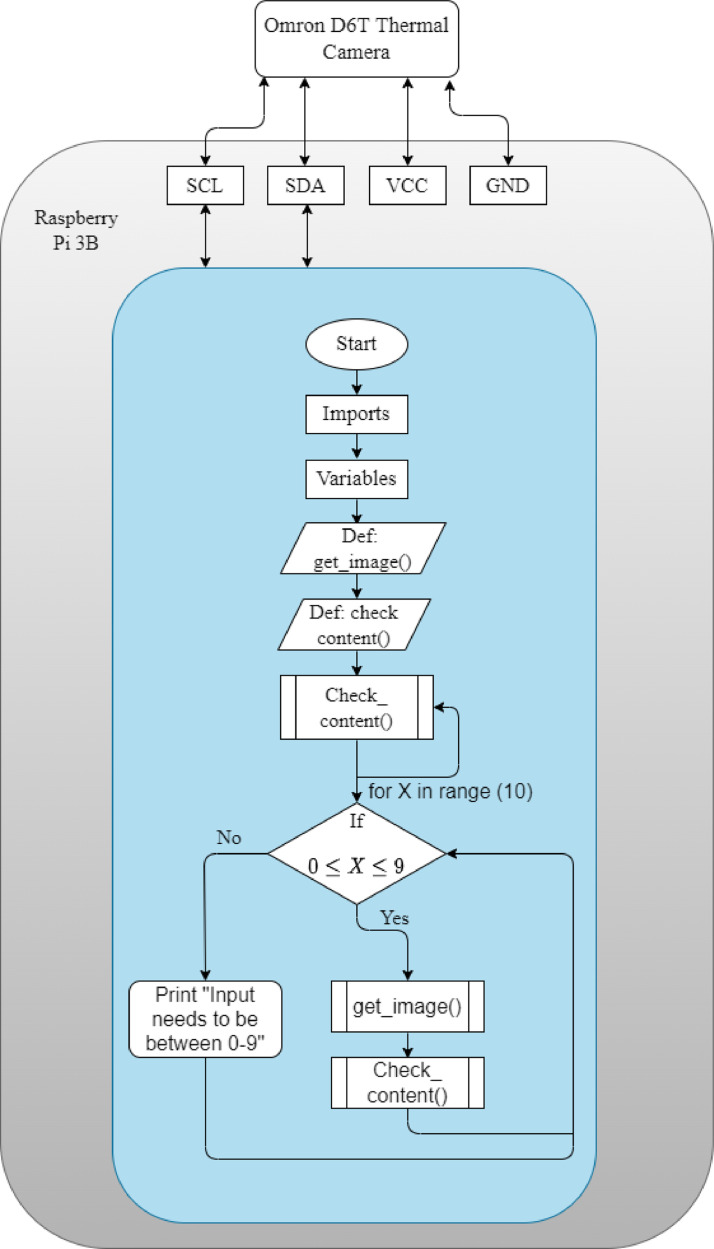
Procedure for capturing thermal images.

The data is captured through the Omron D6T module, using a Raspberry Pi (RPi) 3 Model B as control and storage unit [4], [5], [6]. The camera is attached to the power and ground pins on the RPi. Further, it is attached to the serial data and serial clock pins for data transfer and synchronization. The software program was designed to capture the images based on the number that is being pressed as input between 0 and 9. For example, if the person beneath the camera was showing the number 2 in sign language. The control of the software program would enter 2 as input. The program would then save the images in a folder corresponding to the digit. The detailed procedure of capturing the images is described in Fig. 5.

## Ethics Statement

The data consists solely of hand gestures and contains no personal information. It was a free-for-all campaign, and people gave the hand gestures at their own discretion.

## CRediT authorship contribution statement

**Sreenivasa Reddy Yeduri:** Writing – original draft, Writing – review & editing, Conceptualization. **Daniel Skomedal Breland:** Methodology, Software, Data curation, Visualization, Investigation. **Simen Birkeland Skriubakken:** Methodology, Software, Data curation, Visualization, Investigation. **Om Jee Pandey:** Writing – review & editing, Supervision. **Linga Reddy Cenkeramaddi:** Conceptualization, Supervision, Validation, Writing – review & editing.

## Declaration of Competing Interest

The authors claim than there is no influence from known competing financial interests or personal relationships which have, or could be perceived for the work reported in this article.
